# Innate Lymphoid Cells in Protection, Pathology, and Adaptive Immunity During Apicomplexan Infection

**DOI:** 10.3389/fimmu.2019.00196

**Published:** 2019-02-28

**Authors:** Daria L. Ivanova, Stephen L. Denton, Kevin D. Fettel, Kerry S. Sondgeroth, Juan Munoz Gutierrez, Berit Bangoura, Ildiko R. Dunay, Jason P. Gigley

**Affiliations:** ^1^Molecular Biology, University of Wyoming, Laramie, WY, United States; ^2^Veterinary Sciences, University of Wyoming, Laramie, WY, United States; ^3^Microbiology, Immunology and Pathology, College of Veterinary Medicine and Biomedical Sciences, Colorado State University, Fort Collins, CO, United States; ^4^Institute of Inflammation and Neurodegeneration, Otto-von-Guericke Universität Magdeburg, Magdeburg, Germany

**Keywords:** innate lymphoid cells (ILC), IL-12 family, IFN-gamma, IL-17, apicomplexan parasites

## Abstract

Apicomplexans are a diverse and complex group of protozoan pathogens including *Toxoplasma gondii, Plasmodium* spp., *Cryptosporidium* spp., *Eimeria* spp., and *Babesia* spp. They infect a wide variety of hosts and are a major health threat to humans and other animals. Innate immunity provides early control and also regulates the development of adaptive immune responses important for controlling these pathogens. Innate immune responses also contribute to immunopathology associated with these infections. Natural killer (NK) cells have been for a long time known to be potent first line effector cells in helping control protozoan infection. They provide control by producing IL-12 dependent IFNγ and killing infected cells and parasites via their cytotoxic response. Results from more recent studies indicate that NK cells could provide additional effector functions such as IL-10 and IL-17 and might have diverse roles in immunity to these pathogens. These early studies based their conclusions on the identification of NK cells to be CD3–, CD49b+, NK1.1+, and/or NKp46+ and the common accepted paradigm at that time that NK cells were one of the only lymphoid derived innate immune cells present. New discoveries have lead to major advances in understanding that NK cells are only one of several populations of innate immune cells of lymphoid origin. Common lymphoid progenitor derived innate immune cells are now known as innate lymphoid cells (ILC) and comprise three different groups, group 1, group 2, and group 3 ILC. They are a functionally heterogeneous and plastic cell population and are important effector cells in disease and tissue homeostasis. Very little is known about each of these different types of ILCs in parasitic infection. Therefore, we will review what is known about NK cells in innate immune responses during different protozoan infections. We will discuss what immune responses attributed to NK cells might be reconsidered as ILC1, 2, or 3 population responses. We will then discuss how different ILCs may impact immunopathology and adaptive immune responses to these parasites.

## Introduction

Apicomplexa are a large family of protozoan parasites, which are obligate intracellular parasites of warm-blooded animals. Almost all of them are considered to be major health threats to humans and livestock throughout the world. These include but are not limited to *Toxoplasma gondii* (*T. gondii*), *Plasmodium* spp., *Cryptosporidium* spp., *Eimeria* spp., and *Babesia* spp. Others do exist, but this review will focus on the genera listed above. They can be generally divided into either vector borne or orally transmitted pathogens. Apicomplexans have reduced genome sizes compared to higher eukaryotes, but they encode several different types effector proteins that allow them to develop a very complex relationship with their hosts and contribute to virulence. The vector borne apicomplexans include the mosquito borne *Plasmodium* spp. and the tick borne *Babesia* spp. Orally infectious apicomplexans include *T. gondii, Cryptosporidium* spp. and *Eimeria* spp. *Plasmodium* spp. infects ~200 million people and kills around 400,000 a year (1). *Babesia* spp. is a newly emerging parasitic infection of humans (2, 3). *Toxoplasma gondii* infects ~30% of people worldwide and is the third leading cause of food borne illness in the U.S (4). There are on average 750,000 new cases of *Cryptosporidium* spp. per year in the U.S. alone and the parasite is distributed worldwide (5). *Eimeria* spp. infections can be devastating to chicken and beef farms, but it does not appear to be infectious to humans (6). Many of these protozoan parasites can be problematic for people with compromised immune systems especially those with HIV/AIDS. Moreover, in immune competent individuals the majority of these infections can cause considerable tissue morbidity and pathology resulting in long term damage to the host. In the case of *T. gondii* infection there is increasing evidence that persistent infection could contribute to psychiatric disorders and neurodegenerative disorders (7). Thus, gaining a better understanding of the immune factors involved in control of these pathogens as well as the factors that contribute to immunopathology is important to reduce negative health outcome caused by these common infections.

Immune control of apicomplexans largely depends upon induction of adaptive immunity via a T helper type 1 (Th1) response and production of IFNγ (8). In addition to Th1 response, IL-17 production and associated inflammation also are induced (9–12). In many cases this Th17 response appears to contribute to immune pathology associated with these infections. In order to develop either a Th1 or Th17 response, innate immune cells have to be triggered to produce the cytokines important in directing which types of T helper responses develop. In comparison to viral infections where much is known about innate immune cell composition and how these cells function in protection and immunopathology, less is known in the context of apicomplexan infection. Active areas of research to expand this knowledge in protozoan infection exist including an understanding of how innate immune responses contribute to control, cause pathology and influence the development of adaptive responses. However, a major gap in knowledge still exists in understanding all of the innate immune cell populations that are recruited and activated during protozoan infections and what role they each have in protection, causing pathology and/or regulating adaptive immune responses.

**Figure 1:**
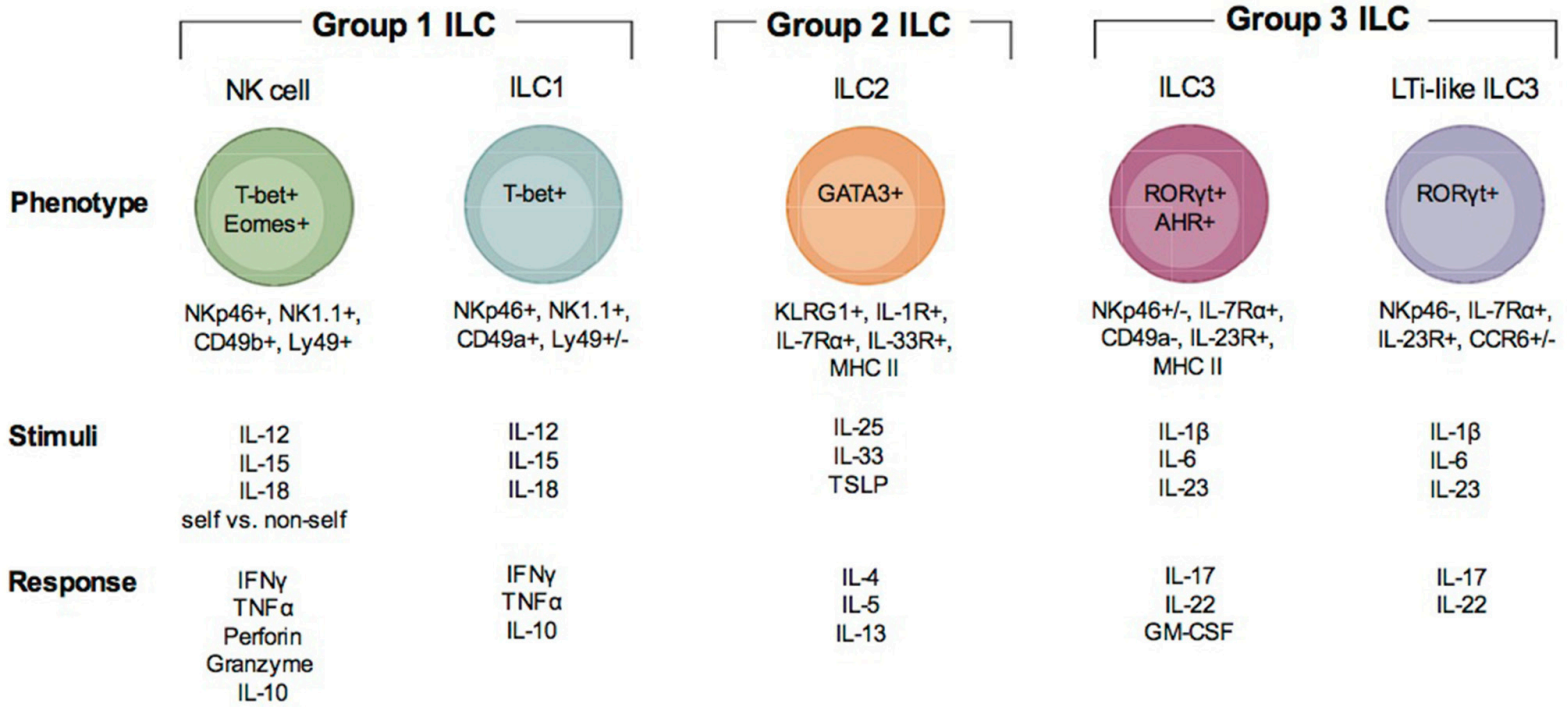
Description of ILC subsets. There are three groups of ILC, group 1 ILC, group 2 ILC, and group 3 ILC. Within each of these groups, subsets of cells are indicated (group 1: NK cells and ILC1; group 2: ILC2; group 3 ILC3 and LTi-like ILC3). Each ILC is illustrated with the transcription factors important for their development and function, their surface phenotype, the stimuli that is known to activate them and the immune factors produced when they are activated and responding to infection.

Innate immune responses are critical in setting the stage for how the adaptive immune system responds to infection. Many types of cells of either myeloid or lymphoid origin within the innate immune cell compartment contribute to this process. Common myeloid progenitor derived cells include, granulocytes, monocytes/macrophages, dendritic cells, and mast cells (13). These myeloid populations initiate a response to infection and activate the lymphoid cell populations by producing chemokines and cytokines, presenting antigen, and providing costimulation. Innate immune cells are derived from the common lymphoid progenitor and were originally only thought to include Natural Killer cells and some innate B cell like populations. However, in 2013 after a continuous flow of new discoveries about innate immune responses by lymphoid derived cells, the newly appreciated complexity of lymphoid progenitor derived innate immune cells was acknowledged and the Innate Lymphoid Cell classification was proposed (14). As a result, NK cells were formally recognized to not be the only cell comprising this population and there exist 3 groups of innate lymphoid cells (ILC) (Figure 1). Group 1 ILC include what are now considered to be conventional NK (NK) cells and ILC1 (15). Currently, group 2 ILCs include ILC2s, and group 3 ILC include ILC3 and Lymphoid Tissue inducer like cells (LTi-like ILC3) (16, 17). Conventional NK cells appear to be the only cytotoxic cell while all the other ILCs follow the pattern of helper CD4 T cells and produce cytokines and other soluble factors that help adaptive immune responses develop. Conventional NK cells have been studied for years in apicomplexan infection and their importance in producing IFNγ during acute infection is very well-established (1, 4). However, several studies demonstrate that what were considered to be NK cell responses during parasitic infection might be responses of other ILCs to infection. Given the updated view of the diversity of ILC populations, a major gap in knowledge in the apicomplexan field is how do different ILC populations contribute to innate and adaptive immunity and/or immunopathology associated with these infections. Another important question to address is whether and how ILC populations positively and negatively regulate adaptive immunity to apicomplexans. Where published data is available, we will detail what is known about the development, activation, and effector functions of NK cells and other ILCs in the context of different apicomplexan infections. We will also discuss the possible roles of non-NK cell ILC populations in protection or pathology associated with the different apicomplexan infections and how they may impact adaptive immunity during infection with these parasitic protozoans.

## Group 1 ILC

Group 1 ILCs include the conventional NK cell and ILC1 (Figure 1). NK cells have been extensively studied in the context of Apicomplexan infection (1, 4, 18, 19). ILC1 have only recently been investigated (20). Group 1 ILCs can be identified by surface expression of the natural cytotoxicity receptor NKp46 and NK1.1 (only in mice that express *NKrp1*). ILC1 can be distinguished from NK cells by their surface expression of very late antigen 1 (VLA-1) or CD49a and TNF—related apoptosis inducing ligand (TRAIL) (15). In the resting state, NK cells are negative for CD49a and positive for very late antigen 2 (VLA-2) or CD49b. Although TRAIL is considered a marker specific for ILC1s, evidence supports that it is also expressed by immature NK cells (iNK) prior to their maturation in the mature NK cells (mNK) which are TRAIL negative (21). NK cells can be found in many tissues and are continuously circulating through the blood. ILC1s are considered to have tissue residence and have been found in both mucosal and non-mucosal tissues (15). These include the spleen, liver, salivary glands, peritoneal cavity, gut, and uterus. NK cells are dependent on expression of T-bet and Eomes for their development and function (21, 22). ILC1 are only dependent upon T-bet (15). Due to the apparent plasticity in ILC populations, ILC1-like cells can arise from both ILC2 and ILC3 (23). Transdifferentiation of ILC2 and ILC3 into ex-ILC2 and ex-ILC3 ILC1 are marked by their increase in NKp46, NK1.1, CD49a, and T-bet expression. These types of ILC1 are also positive for the high affinity IL-7 receptor subunit alpha (IL-7Rα) or CD127 (23). NK cells are the only group 1 ILC that can be cytotoxic. NK cell cytotoxicity is important for killing virally infected cells and tumor cells. The importance of NK cell cytotoxicity in apicomplexan infection is still unclear. NK cells and ILC1 and ex ILC2 and 3 ILC1s produce high levels of IFNγ and TNFα in response to Th1 inducing cytokines IL-12, IL-15, and IL-18 (15). Via their IFNγ production they help control apicomplexan infection.

## Group 2 ILC

Group 2 ILC includes ILC2 (Figure 1) (16). ILC2 could be involved in apicomplexan infection, however, their importance is still not well defined (24, 25). An important distinction between ILC2 and other ILCs is that to date a distinct surface marker has not been identified. ILC2 are lineage (CD3, CD19), NKp46, and NK1.1 negative and CD127, c-Kit(CD117), KLRG1, and the IL-33 receptor (ST2) positive (16, 26). The ILC2 is tissue resident similar to ILC1 and is found at mucosal tissues including the intestine and lungs. ILC2 development and activation depend on the transcription factor GATA3 and they contribute to Th2 responses by producing IL-4, IL-5, IL-9, and IL-13. ILC2 can also express MHC Class II and may be able to prime CD4 T cells (16). ILC2 are known for their importance in immunity against helminth infections to promote tissue repair. Since they express the IL-33 receptor ST2 they can sense tissue damage and respond to promote tissue repair. They are also damaging as they can contribute to allergic inflammation and asthma. As mentioned above, ILC2 demonstrate a high level of plasticity (27–30). IL-1β and IL-12 can drive them to differentiate into an ILC1-like cells. Thus, in addition to their importance in Th2 responses, when given the proper signals they can produce IFNγ and contribute to Th1 dependent immunity. Therefore, ILC2 could contribute to immune protection and immune system regulation during apicomplexan infections.

## Group 3 ILC

Group 3 ILCs include ILC3 and LTi and LTi-like ILC3 (Figure 1) (17). Recent studies suggest that ILC3 can contribute to immunity during apicomplexan infections, however, much is not known about how these cells impact immunity against these parasites (31). Based on surface phenotype ILC3 can be either positive or negative for NKp46 (17). They are also positive for CD127, CD117, and receptors for IL-1(IL-1R) and IL-23 (IL-23R). LTi and LTi-like-ILC3 are NKp46 and CD49a negative, but positive or negative for CCR6 depending on the tissue in which they reside (32). Some LTi cells can also be CD4 positive and ILC3 can express MHC Class II. Group 3 ILC have a wide tissue distribution and reside in mucosal tissues and their associated lymphoid organs. ILC3 and LTi-like-ILC3 differentiation and function depend on the transcription factors RORγt and the aryl hydrocarbon receptor (AHR). Given their tissue residency they are poised to respond to different environmental cues to either maintain barrier homeostasis or provide an inflammatory response against infection. In response to IL-1β and IL-23, ILC3 produce IL-17A, IL-22 and GM-CSF and LTi-like-ILC3s produce IL-17F and IL-22. LTi-like ILC3 also can produce lymphotoxin α/β (LTα/β) to promote lymphoid tissue development. Since group 3 ILC can produce IL-17 and IL-22, they could contribute significantly to Th17 responses observed in many apicomplexan infections, yet their importance is unclear.

## ILC plasticity

The border between ILC subtypes has become more defined, however as noted above, ILC are highly plastic and can convert into each other depending on the environment they experience (23). For example, under certain inflammatory conditions, ILC2 and ILC3 can express T-bet and produces Th1 cytokines (27, 29, 33). When the conditions permit, these newly generated ILC1 can convert back into ILC2 and ILC3. This cellular plasticity is likely essential for the generation of optimal responses against pathogens and maintenance of tissue integrity. Due to this new appreciation of ILC diversity, how different ILC populations participate in immunity to apicomplexan infection has not been well defined. We will next discuss what is currently known about ILCs during these parasitic infections and highlight situations where different ILCs may be involved. We will also discuss how different ILCs could be implicated in adaptive immunity to these pathogens.

## ILC and *Toxoplasma gondii*

**Figure 2:**
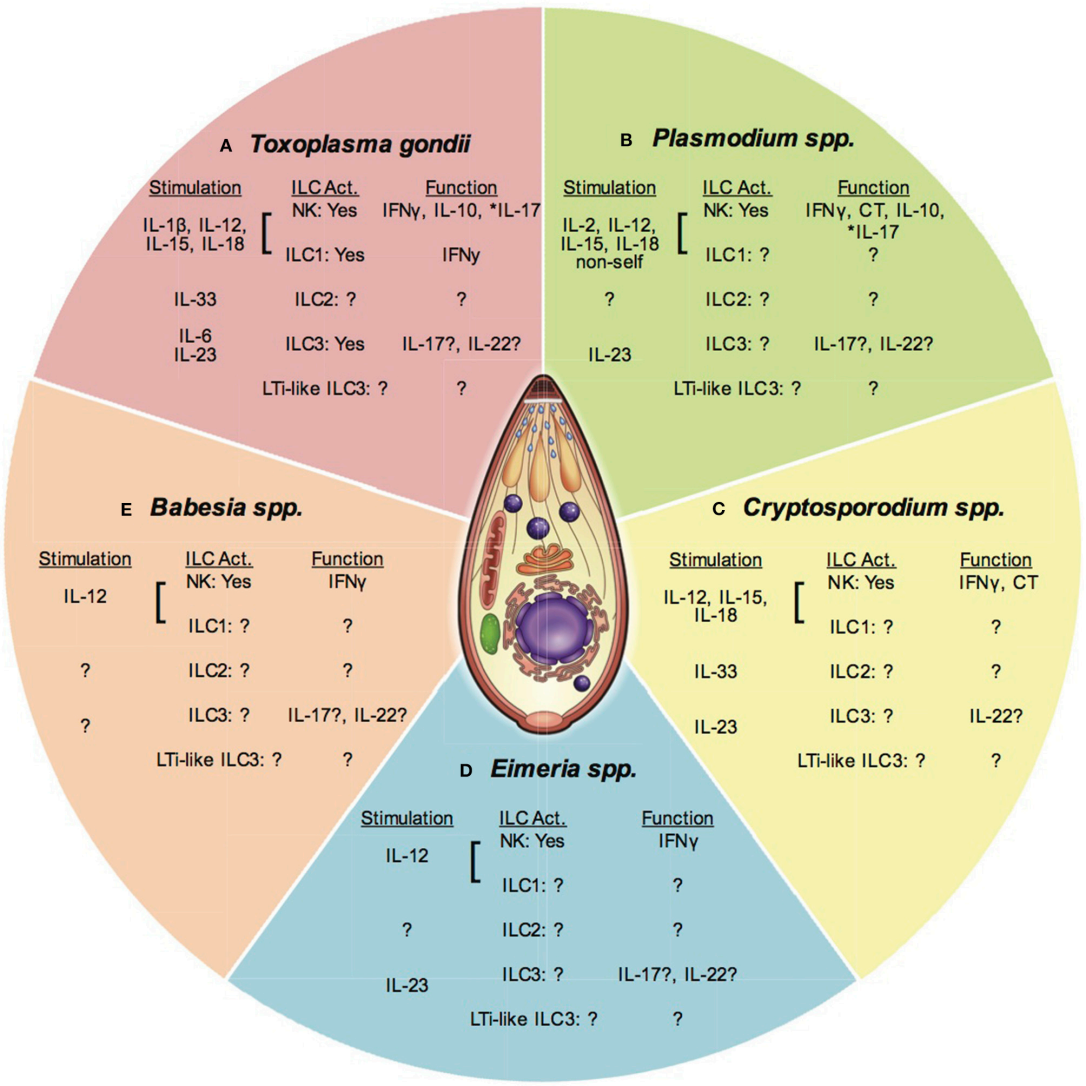
ILC and apicomplexan infection. This figure presents an overview of ILC responses to different apicomplexan infections covered in this article. Each section represents one parasitic protozoan. Under each genus heading there are listed 3 subheadings indicating; (1) Stimuli for each ILC subset (stimulation), (2) The ILC subpopulation activated (ILC Act.), and (3) The function of the activated ILC subset. Question marks indicate where there is no or limited data available. An ^*^denotes where function attributed to NK cells may be from a different ILC population. **(A)** *Toxoplasma gondii* stimulates the production of IL-1β, IL-12, IL-15, and IL-18 that activate NK cells and possibly ILC1 to produce IFNγ and IL-10. IL-17 produced by NK cells may also be produced by other ILC. IL-33 is produced and may activate ILC2. IL-6 and IL-23 are produced and could activate ILC3 for IL-17 and IL-22 production. The importance of LTi-like ILC3 are not known. **(B)** *Plasmodium spp*. stimulates IL-2, IL-12, IL-15, and IL-18, which activate NK cells to produce IFNγ. Recognition of non-self may stimulate NK cell cytotoxicity (CT). These cytokines also stimulate NK cells to produce IL-10. NK cell IL-17 may also be produced by other ILC. The role of ILC2, ILC3, and LTi-like ILC3 are not clear. **(C)** *Cryptosporidium* spp. infection stimulates the production of IL-12, IL-15, and IL-18. These cytokines can activate NK cells to produce IFNγ. NK cell cytotoxicity is also increased after infection, but the stimulus is not known. The importance of ILC1, ILC2, ILC3, and LTi-like ILC3 has not been tested. **(D)** *Eimeria* spp. infection induces IL-12 production that activates NK cells to produce IFNγ. The importance of ILC1, ILC2, ILC3, and LTI-like ILC3 are not known, however evidence suggests IL-17 and IL-22 are produced during infection highlighting the potential activity of non-NK cell ILCs. **(E)** *Babesia* spp. infection stimulates the production of IL-12, which activates NK cells to produce IL-12. The importance of other ILCs has not been investigated at this time.

Even though the ILC classification was recently established to define the innate immune cells of lymphoid lineage, the importance of ILC function for control of *T. gondii* infection has been investigated for many years (4, 34) (Figure 2). Infection with *T. gondii* begins after ingestion of oocysts from cat feces or bradyzoite containing tissue cysts from undercooked meat (4, 8). Acute infection is followed by chronic infection in the CNS and muscle for the life of the host. Innate immune responses at mucosal sites and in secondary lymphoid organs are critical for early control of the parasite. In early studies NK cells were shown to be activated by *T. gondii* infection to be cytotoxic (35). Later on NK cells were shown to be a non-T cell source of IFNγ and were essential for innate immunity to *T. gondii* infection (36, 37). Whether NK cell cytotoxicity is important for early control of *T. gondii* infection is not known and had not been thoroughly tested (4). Importantly, these early studies were some of the first to demonstrate the importance of the IL-12/IFNγ axis in development of Th1 biased immunity (36–38). Indeed IL-12 is required for activation of NK cells to produce IFNγ during *T. gondii* infection (37). Additional cytokines can help stimulate NK cell activation including IL-1β and IL-18 (39, 40). Whether recognition of self-vs. non-self is an important stimulant for NK cells during *T. gondii* infection is not clear (41). To date there have been no observed dominant NK cell populations based on NK cell receptor expression in mice that arise during acute infection suggesting their response is mostly to inflammatory cytokines. Verification of the importance of NK cells in protection against *T. gondii* infection was tested using lymphocyte deficient animals including RAG knockout and SCID mice. In addition NK cell antibody depletion regimes *in vivo* targeting asialo ganglio-N-tetraosylceramide (anti-ASGM1) or anti-NK1.1 were used. These studies laid the foundation for the importance of NK cells in control of *T. gondii* infection.

More recently, studies have addressed how NK cells are involved in immunity against *T. gondii* infection through their impact on other immune cells. NK cell IFNγ can help prime CD8 T cells in the absence of CD4 T cell help (42). NK cell IFNγ can also help activate CD4 T cells during acute *T. gondii* infection (43). Infection of TAP1^−/−^ mice results in reduced CD4 T cell IFNγ production. Adoptive transfer of IFNγ+ but not IFNγ- NK cells restore this deficient CD4+ T cell response. NK cell activity during early during infection could also impact the myeloid cell compartment (44–46). NK cells may enhance DC maturation via NKG2D on the NK cell resulting in more robust CD8 T cell priming during acute infection (44). NK cell IFNγ may be required for the loss of resident mononuclear phagocytes followed by recruitment of circulating monocytes that locally differentiate into macrophages and monocyte derived DC (MoDC) (45). These MoDC then serve as the main source of IL12p40 at the site of infection, which in this study was the peritoneum. Early NK cell (CD49b+CD49a-CD45+TCRb-NK1.1+) IFNγ production during *T. gondii* infection has also been shown to educate the myeloid compartment in the bone marrow. The IFNγ generated by NK cells in the bone marrow skewed monocyte development at that site into a more regulatory phenotype (46). In summary, NK cells via their IFNγ production are able to both positively and negatively regulate other immune cells during *T. gondii* infection. The impact of NK cells on other immune cells could therefore positively or negatively impact the generation of adaptive immune responses to the parasite.

A small number of studies have investigated whether parasite infection of NK cells affects their behavior (47–49). NK cells can be parasitized, however, this occurs at a very low frequency *in vitro* and *in vivo*. These infected NK cells display a hypermotility phenotype and defective function. A recent study indicates that infected NK cells do not contribute to parasite dissemination in the mouse (47). Thus, how direct parasite infection of NK cells impacts the disease course is not known and needs to be further explored.

A major question that now needs to be considered based on the increase in knowledge about additional ILC subsets is can the observations discussed above involve other ILC populations during *T. gondii* infection? This question arises because for many of these studies, the importance of NK cells was further demonstrated by using anti-NK1.1 or anti-ASGM1 to deplete the cells *in vivo* (12, 36). These treatments could easily target other ILC types because of their expression of NK1.1 and or asialo GM1 on their surfaces. One population of ILC that could also be involved in parasite control and in shaping the immune response to the parasite is the ILC1. ILC1 are tissue resident cells that produce large amounts of IFNγ (15). Support for this idea was demonstrated by a study that identified the common helper innate lymphoid progenitor cell or CHILP (20). In these studies, ILC1 as defined by their phenotype Lin-NKp46+NK1.1+Tbet+Eomes- in the small intestine produced the highest amount of IFNγ compared to NK cells and NKp46+NK1.1+ ILC3. Using T-bet deficient (Tbx21^−/−^) mice to eliminate ILC1 development, ILC1 IFNγ was significantly reduced and parasite burdens were significantly increased in the gut. These results suggest that ILC1 resident in specific tissues can also influence the outcome of *T. gondii* infection. A good example that different ILC subsets are involved in different tissues was shown by a separate study using the same T-bet deficient animals (50). In this study the authors found that the NK cells still produced a high level of IFNγ in response to *T. gondii* in the spleen in the absence of this transcription factor. These results demonstrate that in different tissues different transcription factors are important for ILC (NK cell vs. ILC1) responses to *T. gondii*. They also suggest the potential critical role of Eomes and not T-bet in development of NK cell responses in the spleen. Thus, where group 1 ILC IFNγ is impacting immunity to *T. gondii* further investigation is needed to distinguish between NK cells and ILC1 as the source of this cytokine and in what tissues they are working.

Other ILC populations may also be playing an important role in immunity to *T. gondii*. These include the ILC2, ILC3, and LTi-like ILC3 populations. Although ILC2 are helper cells that drive Th2 responses, they could be important during *T. gondii* infection. ILC2 could have a role in dampening the inflammatory response to *T. gondii* infection. ILC2 respond to tissue damage at mucosal sites associated with parasitic helminth infections (16). Their response is controlled by alarmins including IL-33 and IL-1β. Interestingly, a previous report using ST2 (IL-33R) deficient mice demonstrated that these mice were more susceptible to developing inflammatory lesions and Toxoplasmic encephalitis associated with increases in iNOS, IFNγ, and TNFα (25). However, a separate study demonstrated that IL-33 and ST2 correlated with greater immunopathology, inflammation and ocular toxoplasmosis (51). Therefore, whether ILC2 are important or not is unclear. It is still possible that as a result of mucosal tissue damage caused by acute *T. gondii* infection, the release of IL-33 and signaling induced via ST2 could dampen the Th1 biased inflammatory response to the parasite by activating ILC2 to produce Th2 biased cytokines. Whether ILC2s are an important cell type involved as a negative regulator of inflammation and T cell responses during *T. gondii* infection has not been tested and would be important to address in future studies.

Group 3 ILCs could also be an important immune cell involved in *T. gondii* infections, however, very little is known about them. Group 3 ILCs include LTi-like ILC3 and ILC3, both important cell types that are tissue resident and present in several tissues including the gut. They are important for maintaining tissue homeostasis at these sites, but can also promote tissue damage when they are highly activated (17). ILC3 production of IL-22 is thought to help maintain tissue integrity while ILC3 derived IL-17 can be inflammatory and associated with pathology. ILC3 are thought to be resident at mucosal barriers, but are also found in the spleen (32). During *T. gondii* infection a study revealed that IL-17 was produced and signaling through the IL-17 receptor was important for protection by stimulating neutrophil recruitment (52). A separate study demonstrated that IL-17 via IL-17 receptor signaling caused gut immunopathology associated with infection and was important in promoting chronic *T. gondii* infection (53). At this time, the cellular source of IL-17 was not known. One report suggested that NK cells were the source of *T. gondii* induced IL-17 (12). In this study IL-17 levels increased in infected Rag1^−/−^ mice and anti-ASGM1 treatment significantly reduced the levels of this cytokine in these animals. IL-17 production was induced by IL-6, followed by IL-23 and TGFβ, and suppressed by addition of Th1 cytokines (IL-12, 15, and 27). The splenic NK1.1+CD3- cells that produced IL-17 were not secreting IFNγ as no double positive IFNg+IL17+ NK1.1+CD3- cells were detected. These results suggested that this NK cell population might be distinct from the NK cell population involved in early control of the parasite. Taking into consideration the experimental approaches used to define that these cells were NK cells (Rag1^−/−^ and anti-ASGM1) and that these same approaches can target ILC3 may suggest that the ILC3 populations were the IL-17 producers in these studies and not NK cells. Further support for this idea was revealed because the ILC population studied expressed IL-6Ra and the transcription factor RORγt rather than T-bet (12). Group 3 ILC are now known to depend upon expression of RORγt (17). Since ILC3 are also found in the gut, additional studies indicate that an NKp46+ ILC cell develops in the lamina propria and in response to IL-18 recruits inflammatory monocytes into the gut that increase immunopathology associated with *T. gondii* infection (54). A recent study has investigated more specifically the role of ILC3 in the gut during *T. gondii* infection using aryl hydrocarbon receptor deficient mice and demonstrates that these cells appear to negatively regulate T cell activity (31). Thus, again depending on the tissue investigated, ILC3 may have multiple roles including causing disease pathology and potentially as a negative regulator of adaptive immune responses during *T. gondii* infection. Many questions remain unanswered about these cells during this infection including formal dissection of whether ILC3 could positively or negatively impact development and maintenance of T cell responses against *T. gondii*. In addition, ILC3 and LTi-like ILC3 plasticity would be important to investigate during *T. gondii* infection.

As discussed above, ILCs may also play a role as negative regulators of immunity against *T. gondii*. Using systemic *T. gondii* as a model infection, NK cells were shown to be capable of producing IL-10 (55). These IL-10 producing cells were defined as conventional NK cells because they were lineage negative (CD3-CD19-TCRβ-) and NK1.1 positive. These cells were also CD127 negative suggesting that they were not ILC2 or ILC3. This regulatory NK cell population was induced by systemic inflammation, and IL-12 and NK cell IL-10 limited IL-12 production by DC. These cells were found to produce IL-10 in lung, liver, brain, blood, but not in spleen and MLN during acute *T. gondii* infection. In another study, NK cells (CD3-CD19-DX5+NK1.1+) were shown to be the major source of IL-10 in spleen, PEC, liver during acute infection (56). Both IL-12 and aryl hydrocarbon receptor (AHR) were required for maximal IL-10 production by these cells. These regulatory NK cells also expressed T-bet, KLRG1 and co-produced IFNγ. The presence of the IL-10 producing NK cells reduced the ability of the mice to control *T. gondii* infection. These data suggest that IL-10 producing cells are present during acute *T. gondii* infection, could be members of group 1 ILC and are important for regulating adaptive immune responses to *T. gondii*. A major open question still is where do these cells originate, what are the long term affects of these cells on T cell responses to the parasite and could they have a negative impact on chronic toxoplasmosis.

There are still many open questions about the roles of different populations of ILCs and *T. gondii* infection. The importance of each ILC subtype has not been fully addressed and there are questions about NK cells that will have to be reinvestigated because of the increase in knowledge of the different ILC subsets in the context of infection. Many studies investigating ILCs during *T. gondii* infection have focused on the acute stage of infection. Their importance in long-term control of the parasite is still not clear especially in chronic *T. gondii* infection and the CNS, which is the current focus of our laboratory.

## ILC and Plasmodium

As with *T. gondii* infection, investigations of ILCs in *Plasmodium* spp. infection have been ongoing for many years. Of the ILCs researched the majority of data has been generated from NK cell specific studies [reviewed in (1)]. Infection with *Plasmodium* spp. in humans begins with the injection of sporozoites of the parasite from the salivary gland of the mosquito into the blood stream of the host (57). Once the parasite is inside the host, it migrates to the liver where hepatocytes are infected. The parasite transforms as it replicates into the merozoite stage, which is released from the infected hepatocyte after lysis of the cell. The merozoites then infect erythrocytes (RBC) and develop into male and female gametes and the cycle is repeated. The innate immune response is important in early control of the parasite in liver, periphery, and secondary lymphoid structures where many innate immune cells including ILCs reside. Inflammation generated by the innate immune response including ILC may contribute to *Plasmodium* spp. pathogenesis and pathology such as cerebral malaria (58). NK cells are critical immune cell type in early and continuous control of *Plasmodium* spp. infections in both mouse models of infection and humans (1) (Figure 2). NK cells and/or other ILC types may also be pathogenic by contributing to the development of cerebral malaria. Additionally, the diverse life stages and tissue locations of the parasite likely require the involvement of distinct ILC subsets. Much is still not known about how each ILC type contributes to these processes during *Plasmodium* spp. infection.

In mice splenic, hepatic and peripheral NK (NK1.1+) cells protect against early stages of malaria infection by producing IFNγ and TNFα (59–62). After anti-ASGM1 treatment and NK cell depletion there was a decrease in IFNγ production and an increase in parasitemia in mice (59, 60). In humans, NK cells are thought to be some of the first cells to produce IFNγ during infection (1, 58). Human NK cells (CD56+) produce IFNγ and TNFα after *Plasmodium falciparum* infection (63–65). Human NK cells (can produce IFNγ after stimulation with *Plasmodium* infected erythrocytes *in vitro* (66). In addition to IFNγ production, peripheral blood NK cells are thought to be stimulated to be cytotoxic in response to parasite infection (67, 68). Human NK cells release cytotoxic molecules when cultured with infected hepatocytes and erythrocytes *in vitro*. NK cells have been observed to directly interact with infected erythrocytes forming conjugates (66, 69, 70). Human NK cells have been shown to kill infected erythrocytes (71). Whether NK cell specific cytotoxicity is important in controlling the parasite *in vivo* is still unclear and still needs to be formally tested in mouse models of infection or in humans.

NK cell activation during *Plasmodium* spp. infection is mediated by several signals including cytokines produced by other immune cells and potentially via self-vs. non-self-recognition. Generally, the classic IL-12/IFNγ axis applies to this infection as well as it does to other Apicomplexan infections. Studies are consistent in showing that IL-12 is essential for IFNγ production by NK cells during *Plasmodium* spp. infection (72). Not only is IL-12 important for activation, but also there is interplay between several cytokines and the activation of NK cells to produce IFNγ in response to IL-12. IL-18 in combination with IL-12 can enhance NK cell IFNγ production in mice in response to *Plasmodium* spp. infection (73). This is via IL-18 dependent up regulation of CD25 (IL-2Rα) expression on NK cells allowing them to be more sensitive to IL-2 and produce IFNγ. This IL-18-IL-12-IL-2-NK cell IFNγ is thought to also be occurring in humans exposed to *Plasmodium* spp. infection (65, 74, 75). IL-2 produced by antigen-specific CD4 T cells augmented NK cell activation in immunized individuals. These human studies also demonstrated that different individuals had variable NK cell activation after exposure to infected erythrocytes (75). One hypothesis is that the variability in human NK cell responses is caused by polymorphisms in KIR and/or HLA genes. IL-15 is another cytokine important in NK cell development and function. The role of IL-15 in NK cell activation during *Plasmodium* spp. infection is less clear. One study showed that IL-15 enhanced NK cell IFNγ production (76). In another study IL15^−/−^ DC were as good as WT DC in activating NK cell IFNγ production *in vitro* (72). This is similar to a study with *T. gondii* infection that demonstrated IL-15 is dispensable for NK cell activation (77). In response to *Plasmodium* spp. infection, NK cells are also activated by interactions with monocytes and monocyte derived DCs (70, 78). One of the interactions is dependent upon IL-18. Another interaction is dependent upon direct macrophage to NK cell contact. This cell-to-cell interaction is thought to promote NK cell IFNγ production via interaction between LFA-1 on the macrophage and intercellular adhesion molecule-1 (ICAM-1) on the NK cell (79). In regard to cytotoxicity targeted against hepatocytes and erythrocytes, the exact mechanism of NK cell recognition of these cells remains unknown. Several studies addressed whether NK cell expression of ICAM-1, PECAM, VCAM, CD36, CSA, NKp30, NKp44, NKp46, NKG2D, and the expression of PfEMP1 or heat shock protein 70 on infected erythrocytes facilitated this interaction, however, an exact mechanism is still not known and needs further exploration (71, 79–81).

As with *T. gondii* infection, NK cells may also impact the function of other immune cells and development of adaptive immune responses to *Plasmodium* spp. infection. However, very little has been investigated about how NK cells or other ILCs are involved. NK cells may increase DC maturation and cytokine production facilitating T cell priming (72). In one study, after infection with *P. chabaudi* NK cells promoted DC maturation *in vitro*, IL-12 production and ability to prime CD4 T cells to proliferate and produce IFNγ. Another study demonstrated that NK cell activation *in vivo* was not required for DC maturation or DC-mediated priming of CD4^+^ T cells specific for OVA antigens expressed by *P. berghei* ANKA (82). This study demonstrated that NK cells (NK1.1+) contributed to the DC-mediated priming of CD8^+^ T cells *via* a mechanism that required IL-12. Although these studies may differ in mechanism, it appears that similar to *T. gondii* infection, activated NK cells are important for DC priming of adaptive immunity against *Plasmodium* spp. This NK cell dependent enhancement of T cell priming appears to depend upon IFNγ. Whether activated NK cells can take the place of helper T cells in helping the priming of CD8 T cells has not been addressed during *Plasmodium* spp. infection. How NK cell IFNγ could impact development of long-term immunity to *Plasmodium* spp. is also not understood. There are still many questions about the importance of NK cells in their role during *Plasmodium* spp. infection.

To date very little is known about other ILCs and *Plasmodium* spp. infection. Many of the observations about NK cells in malaria could also be attributed to other ILC subsets. Again, this is because NK cell targeting experimental strategies (phenotype: NK1.1+, *in vivo* depletion: anti-NK1.1, anti-ASGM1) also can target the other ILC populations. Evidence supporting the involvement of other ILC in immunity to *Plasmodium* spp. is found in studies elucidating the mechanisms involved in development of cerebral malaria (CM) (83). The first ILC to consider is the ILC1 because of its tissue residency in the liver and ability to produce high levels of IFNγ (84). Although NK cell IFNγ production is important for reducing parasite numbers early during infection, the IFNγ producing liver ILCs could be ILC1. T-bet^−/−^ animals have elevated parasitemia after *Plasmodium berghei* ANKA infection (83). T-bet deficiency would implicate ILC1 as a controller of acute infection because development of ILC1 is T-bet dependent (15). Interestingly, although parasite burden was increased, T-bet deficiency reduced the severity of CM suggesting that T-bet dependent ILC1 development and activation could also cause immunopathology. A recent study indicates that NK cells and ILC1 are lost in peripheral blood of humans infected with *Plasmodium falciparum* and spleens and livers of mice infected with *Plasmodium chaubaudi chabaudi* AS (85). Using NKp46-iCre mice crossed onto myeloid cell leukemia sequence-1 floxed mice (Mcl1) to genetically ablate mature NK cells, there was no difference in parasitemia compared to WT controls. Using NKp46 iCre mice crossed onto TGFβR2 floxed mice to genetically ablate ILC1, again there was no difference in parasitemia. The results of these studies may suggest that early liver control by NK cells and ILC1s is important, but once the parasite reaches the blood, group 1 ILC may be less able to control the infection.

ILCs are not only important for protecting against infection, but they can also cause immunopathology associated with infections (17). As noted above, the data from studies of CM support this hypothesis (58). In CM susceptible C57BL6 mice, experimental CM is characterized by overproduction of Th1 cytokines (IFNγ, IL-12, and TNFα) (83, 86). Therefore, NK cells and ILC1 could promote CM through the production of inflammatory cytokines including IFNγ. NK cell IFNγ production has been shown to help recruit CXCR3+ T cells into the brain (86). T-bet deficient mice survived experimental CM longer but had higher parasite burdens, indirectly suggesting the potential involvement of group I ILC in contributing to CM pathogenesis (83). While group 1 ILC may be both protective and a cause of immune pathology during *Plasmodium spp*. infection, group 2 ILC may help negatively regulate inflammation and thus prevent development of CM. A recent study suggests that ILC2 may contribute to protection against development of CM (24). ILC2 are sensitive to IL-33 via the expression of the IL-33 receptor ST2 (17). Administration of IL-33 prevented development of CM (24). The therapeutic effect of IL-33 was associated with the expansion of ILC2 and their production of IL-4, IL-5, and IL-13. Adoptive transfer of ILC2 into *Plasmodium berghei* ANKA infection mice increased the frequency of alternatively activated macrophages and T regulatory cells and reduced the severity of CM.

To date there have been no published studies on group 3 ILC including ILC3 and LTi-like ILC3 and *Plasmodium* spp. infection. ILC3 can produce IL-17, IL-22 and GM-CSF in response to IL-1β and IL-23 (17, 32). Based on the function of ILC3 there could be support that they are responding during *Plasmodium* spp. infection. Whether they are protective or causing pathology has not been established. However, during malaria infection in mouse experimental models and humans IL-17 levels increase (87–89). In several cases the increased IL-17 was independent of CD4+ Th17 cells. Macrophages may be one source, but another source not measured in this study could be ILC3s (87). Whether IL-17 is protective or pathogenic is not clear because the data from multiple studies is contradictory (9, 87–91). In mice, IL-17 may help in protection because IL-17 KO mice have higher parasitemia (87). However, in a human study looking at the association of inflammation including IL-17 in Plasmodium induced multiple organ dysfunction (MOD) and CM, high levels of IL-17 in patients was associated with the highest level of MOD (89). *Plasmodium* spp. infection of AhR KO mice, which are deficient in ILC3, were more susceptible to CM and generated higher IL-17 and IL-6 in brain (91). Lastly, IL-17 deficient and IL-23 deficient mice developed CM similarly to WT mice and similar levels of parasitemia (87, 90). Another important function of ILC3 is the maintenance of tissue immune homeostasis through IL-22 production. Two independent studies have demonstrated that in the absence of IL-22 (IL-22 KO mice) pathology cause by *Plasmodium* spp. infection is more severe (88, 92). Again, whether this IL-22 is coming from ILC3 is not known. Current information about IL-17 and IL-22 produced during *Plasmodium* spp. infection does not definitively suggest ILC3 are an important cell type for immunity. However, given the lack of ILC3 specific studies performed, their production of these cytokines may make these studies important to explore. LTi-like ILC3 have not been explored in *Plasmodium* spp. infection. Overall, even though a recent study suggests that ILCs are irrelevant (85), there are still substantial gaps in knowledge about ILCs and *Plasmodium* spp. that would be important to investigate.

Another open question that has not been investigated in *Plasmodium* spp. infection is whether and how ILC populations can regulate adaptive immune responses. ILCs can both positively and negatively regulate adaptive immunity. NK cell IFNγ may help prime T cell responses during *Plasmodium* spp. infection (72). During *T. gondii* infection, NK cells and/or other ILC produce IL-10 (55, 56). This NK cell IL-10 may negatively regulate the adaptive immune response against the parasite likely to prevent immunopathology. A recent study has now demonstrated that treatment of mice with an IL-15 complex (IL-15C) stimulates NK cells to produce IL-10 during *Plasmodium berghei* ANKA infection (93). This NK cell IL-10 was required to protect against CM. Whether NK cell or other ILC IL-10 production in response to *Plasmodium* spp. infection has an impact on development of adaptive immunity to *Plasmodium* spp. infection will be important to further explore.

## ILC and *Cryptosporidium*

There is very limited information of the importance of ILCs during *Cryptosporidium* spp. infection (Figure 2). Infection with *Cryptosporidium* spp. occurs via ingestion of oocysts in contaminated water (5). The parasite remains in the small intestine living inside of gut epithelial cells and is a major cause of diarrhea in people. Innate immunity against the parasite is important for control of the parasite, however there is still limited knowledge about the factors that are critical for this response. This is especially important to investigate because of the mucosal barrier location of the infection where NK cell, ILC1, ILC2, ILC3, and LTi-like ILC3 can all be present (15–17). The level of inflammation generated by these cells could have a positive and or negative impact on infection pathology with this parasite. Results from an early study suggested that a non T cell source of IFNγ was important for control of *Cryptosporidium* spp. infection in mice (94). Subsequent studies suggested that NK cells were not involved into the control of infection (95, 96). Anti-ASGM1 treatment of SCID mice to deplete NK cells did not result in increased infection pathology. However, more recent studies indicate that innate lymphoid cells are protective against *Cryptosporidium* spp. infection (97). Both adult Rag2^−/−^ and Rag2^−/−^γc^−/−^ mice developed chronic infection but parasite burdens were higher and intestinal pathology was worse in Rag2^−/−^γc^−/−^ mice, which eventually succumbed to the infection. Interestingly, in contrast to adult mice, neonatal mice of both genotypes were able to survive the infection, however, Rag2^−/−^γc^−/−^ had higher parasite burdens for a more extended period of time as compared to Rag2^−/−^ (18). Neonatal C57BL6 mice treated with anti-NK1.1 were slower in controlling the infection and had higher parasite burdens (97). In Rag2^−/−^γc^−/−^ protection was attributed to IFNγ produced by peritoneal macrophages that were IL-18 and IL-12-dependent (98). Whether NK cell IFNγ is also important for control of *Cryptosporidium* spp. infection is still not clear and needs further investigation. In addition the mechanisms by which NK cells could be activated in *Cryptosporidium* spp. infection have not been thoroughly tested. IL-12, one of the potent activators of NK cell IFNγ production, is produced and is needed for immunity against *Cryptosporidium* spp. in mice (99, 100). In humans, peripheral blood NK cells (CD3-CD16+CD56+) were shown to be cytotoxic against cryptosporidium infected intestinal epithelial cells in the presence of IL15 *in vitro* (101). IL-15 induced increased expression of NKG2D receptor on NK cells and that correlated with increased expression of the NKG2D ligands MHC class I-related molecules MICA and MICB on infected intestinal epithelial cells. This data suggests that there may be direct recognition of the infected epithelium by NK cells during this infection (101). Therefore, it is possible that IL-12 and IL-15 are important NK cell activation signals during *Cryptosporidium* spp. infection. However, this has not been thoroughly tested and whether these signals are critical for parasite control is still unclear.

Beyond this very basic knowledge about NK cells and their involvement in protection against *Cryptosporidium* spp. infection, nothing is known about other ILCs and their role in immunity against infection. In addition it has not been tested whether and how ILCs can (1) impact the function of other immune cells (monocytes, macrophages, DCs and T cells); (2) affect the pathology associated with disease; (3) positively or negatively regulate adaptive immunity to this parasite. A small hint that other ILCs may be involved was discovered in a neonatal lamb infection model of *Cryptosporidium parvum* (102). After infection of neonatal lambs, total NKp46+ cells increased in numbers. The frequency of perforin+ cells increased in NKp46+CD16+ and NKp46+CD16- subsets. In addition, IL-22 mRNA expression was upregulated in small intestines of infected lambs. Whether these NKp46+ cells were ILC3s or other ILC is not known (102). ILC3 could be responding to infection because IL-17 is produced in response to *Cryptosporidium* spp. Infection (10, 103, 104). However, no clear links have been established and more research is needed to dissect the roles of different ILCs in *Cryptosporidium* spp. infection. Elucidating the role of different ILCs in control of *Cryptosporidium* spp. could lead to better therapy and vaccine design to help treat this infection.

## ILC and *Eimeria*

Similar to *Cryptosporidium* spp., very little is known about NK cells and other ILC and their role in protection vs. pathology during *Eimeria* spp. infection. This is a difficult infection to study ILCs because the host animals are chickens and other livestock and thus have limited reagents available. However, this is another important apicomplexan infection that could provide more insight into how the immune system functions in response to other gut tropic apicomplexans. Infection with *Eimeria* spp. occurs via ingestion of fecal matter containing oocysts of the parasite, which then cause severe inflammation in the mucosa of the gut (105). *Eimeria* spp. is a major cause of disease that can impair productivity in livestock including chickens. Similar to other apicomplexan infections, *Eimeria* spp. stimulates a very strong Th1 response that is initiated by innate immune cells that could include ILC (105). Studies investigating innate immunity to this parasite have focused mainly on NK cells and not other ILCs (Figure 2). Early investigations of the importance of NK cells in *Eimeria* spp. infection in mice suggested that NK cells were not involved in providing protection (106). Even though infection of BALBC mice with *E. vermifornis* induced an increase in splenic and mesenteric lymph node (MLN) NK cell cytolytic activity, treatment with anti-ASGM1 to deplete the NK cells did not increase parasite burdens in BALBC mice. A later study demonstrated that in chickens, splenic and intestinal NK cell activity via cytotoxicity as measured by ^51^Cr-release assay decreased after primary infection followed by recovery of this activity (19). However, during secondary *Eimeria* spp. infection, NK cell activity was increased. Secondary intraepithelial lymphocyte derived NK cell activity was accompanied by increase in number of ASGM1-expressing cells. In beige/beige (bg) mice that are NK cell deficient, replication of *E. vermiformis* was reduced (107). A protective role of NK cells in the immune response against *Eimeria* spp. was demonstrated using SCID, SCID/bg and C57BL6 mice treated with anti-NK1.1 (108). After primary infection with *E. papillata*, WT mice depleted of their NK cells with anti-NK1.1 had higher parasite shedding compared to isotype control treated animals. This NK cell dependent protection may have been due to IFNγ production however this was not test directly. The mechanisms underlying the activation of NK cells during *Eimeria* spp. infection most likely involve IL-12 as it is upregulated after infection, however there are no studies that have tested this directly (109). Whether and how other cytokines and signals impact the development of NK cells responses to *Eimeria* spp. infection have not been addressed.

The role of non-NK cell ILCs has not been addressed in *Eimeria* spp. Given the gut pathology that develops in infected animals ILC1, ILC2, and ILC3 (both ILC3 and LTi-like ILC3) may be involved. Based on research into IL-17 and IL-22 production and its importance in this disease implicates ILC3 may be responding to infection (11, 110). In the absence of IFNγ signaling, mice infected with *Eimeria falciformis* had greater body weight loss and gut pathology, but had a lower parasite burden (110). In these animals IL-17A and IL-22 expression was significantly increased. Importantly antibody blockade of IL-17 and IL-22 reduced the pathology associated with infection. This infection pathology was thought to be CD4 T cell dependent because Th17 CD4 T cells expanded in the absence of IFNγ. Whether ILC3 were also producing IL-17 and IL-22 was not tested in this study. A second also demonstrated that IL-17 was a cause of gut pathology in chickens (11). Since ILC3 can help maintain tissue integrity and also cause pathology at mucosal sites, it is possible they contribute to *Eimeria* spp. associated pathology. Outstanding questions are the importance of ILC in protection and immunopathology, impact of ILC on other immune cells function and impact of ILC on positive and negative regulation of adaptive immune responses to *Eimeria* spp. Understanding their role in infection may help to develop therapies to treat this infection.

## ILC and *Babesia*

Knowledge about ILCs and *Babesia* spp. infection is very limited. How ILC function in response to this infection is still not thoroughly explored, however, based on other apicomplexan infections, they could be very important for at least early control of *Babesia* spp. infection (Figure 2). *Babesia* spp. infection of humans begins after the tick *Ixodes scapularis* harboring sporozoites of the parasite has a blood meal from the host (3). Sporozoites infect RBCs where they replicate as trophozoites eventually transforming into merozoites. Blood stage infection causes hemolysis, fever and fatigue in individuals infected. Increasing rates of infection in people have been observed in endemic regions and *Babesia* spp. has severe health consequences for immunocompromised people. Interestingly, this parasite remains in the blood stream during infection and immunity seems to depend upon the spleen as splenectomyzed people are more susceptible to infection (3). NK cell activity was increased in spleen and peritoneal excudate cells (PEC) of infected with *Babesia microti* mice. However, the course of infection in NK cell deficient bg mice was unaltered (111). NK cell frequencies increased in blood, spleen, and liver of BALBC mice infected with *Babesia* spp. (112). Experiments performed in SCID mice on the C57BL6 background indicated that control of *Babesia* spp. was independent of adaptive immune cells (113). Control of *Babesia* spp. in mice was shown to be dependent upon IL-12 and IFNγ signaling because STAT4 and IFNγR2 deficient animals were more susceptible to infection (114). Loss of NK cells from anti-ASGM1 treatment also resulted in elevated susceptibility to *Babesia* spp. Infection (114). *Babesia* spp. is a dangerous pathogen for cattle (3). NK-like cells proliferated in the spleens of young calves during early response to *B. bovis* (115). Bovine splenic NK cells (NKp46+CD3-CD2+/–CD8+/–) produced IFNγ in the presence of supernatants from *Babesia bovis*-exposed monocytes in an IL-12 dependent manner (116). Bovine NK cell IFNγ production required direct cell-to-cell contact with DCs in co-culture after cytokine stimulation (117). Interestingly, bovine NK cells were more cytotoxic when co-cultured with non-cytokine stimulated DCs. Taken together as with other apicomplexans, group 1 ILCs and specifically NK cells play an important role in early control of *Babesia* spp. infection.

Whether other ILCs are responding and playing a role in *Babesia* spp. infection is not known. However, due to the location of this infection (blood and spleen) other ILC may be less important for this infection. Interestingly, *Babesia* spp. appears to predominantly induce a Th1 response as IL-17 and IL-22 levels did not significantly change in a mouse model of infection (2). More in depth investigation of ILC subsets will be needed to fully assess the role of these cells in immunity against *Babesia* spp. infection. This would include studies exploring how ILC can positively and negatively impact adaptive immune responses.

## ILCS and Apicomplexa Conclusions and Future Directions

ILCs are important cells for the early control of Apicomplexan infections via their production of IFNγ (Figure 2). NK cells and possibly ILC1 in many of these infections are the source of this cytokine, which is made in response to IL-12, IL-15, IL-18, and IL-2. Although very few studies have dissected the importance of ILC2 and ILC3 in apicomplexan infection, there are hints that these cells are responding to these infections and could either be protective, helping to dampen inflammation (NK-ILC1-IL-10, ILC2-IL-33, ILC3-IL22) or potentiating inflammatory pathology (NK-ILC1-IFNγ ILC3-IL-17). However, there are still major gaps in knowledge for apicomplexan infections about all of the ways ILC contribute to protection and impact overall immunity to these protozoan pathogens. ILC not only provide early protection, but could also participate in the generation and maintenance of adaptive immune responses against these important parasitic infections. ILCs are important in priming T cell responses either indirectly by maturing APCs or directly via their cytokine production. Immune factors such as cytokines or signaling molecules produced by ILC could have a positive or negative impact on primed T cell long-term fate and memory differentiation. ILC could also contribute to long-term protection by developing memory-like responses to these protozoan pathogens as they do to viral infections (118). Interestingly, during *Plasmodium* spp. vaccination, memory CD4 T cells directed memory-like NK cell responses during secondary infection in vaccinated people (65). How important the ILC contribution to adaptive recall responses during all apicomplexan infections is not well-understood. Lastly, ILC could be cells that are important in honing the adaptive response against these pathogens either making the memory T cell pools better or decreasing their ability to protect against these infections and promoting parasite persistence or susceptibility to reinfection. There are several situations now known during viral infections, in the tumor microenvironment and in autoimmunity where ILC appear to have a negative impact on adaptive immune responses in these different disease situations (4, 119–129). All of these questions will be an important area of research to investigate. Results from future studies of ILC and apicomplexan infections could help improve knowledge of the biology of these complex cells and promote better therapeutic development against these important parasitic pathogens.

## Author Contributions

JG conceived the review. JG, DI, SD, and KF wrote the review. JG, DI, SD, KF, KS, JM, BB, and ID helped edit the review.

### Conflict of Interest Statement

The authors declare that the research was conducted in the absence of any commercial or financial relationships that could be construed as a potential conflict of interest.
